# Habitual physical activity improves outcomes among patients with myocardial infarction

**DOI:** 10.3389/fcvm.2023.1174466

**Published:** 2023-06-12

**Authors:** Sidong Cai, Fangmei Huang, Run Wang, Min Wu, Mingya Liu, Yufen Peng, Gaozhen Cao, Yapin Li, Shuhong Liu, Jiena Lu, Mengqi Su, Yinxia Wei, Kai-Hang Yiu, Cong Chen

**Affiliations:** ^1^ Division of Cardiology, Department of Medicine, The University of Hong Kong-Shenzhen Hospital, Shenzhen, China; ^2^ Division of Cardiology, Department of Medicine, The University of Hong Kong, Queen Mary Hospital, Hong Kong, China

**Keywords:** habitual physical activity, myocardial infarction, major adverse cardiovascular events (MACEs), cardiovascular mortality, cardiac readmission

## Abstract

**Purpose:**

This study evaluates the association between habitual physical activity (HPA) and the outcomes of patients with myocardial infarction (MI).

**Methods:**

Patients newly diagnosed with MI were divided into two groups based on whether they engaged in HPA, defined as an aerobic activity with a duration of no less than 150 min/week, before the index admission. The primary outcomes included major adverse cardiovascular events (MACEs), cardiovascular (CV) mortality, and cardiac readmission rate 1 year following the index date of admission. A binary logistic regression model was applied to analyze whether HPA was independently associated with 1-year MACEs, 1-year CV mortality, and 1-year cardiac readmission rate.

**Results:**

Among the 1,266 patients (mean age 63.4 years, 72% male), 571 (45%) engaged in HPA, and 695 (55%) did not engage in HPA before MI. Patients who participated in HPA were independently associated with a lower Killip class upon admission (OR = 0.48: 95% CI, 0.32–0.71, *p* < 0.001) and a lower prevalence of 1-year MACEs (OR = 0.74: 95% CI, 0.56–0.98, *p* = 0.038) and 1-year CV mortality (OR = 0.50: 95% CI, 0.28–0.88, *p* = 0.017) than those who did not participate in HPA. HPA was not associated with cardiac-related readmission (OR = 0.87: 95% CI, 0.64–1.17, *p* = 0.35).

**Conclusions:**

HPA before MI was independently associated with a lower Killip class upon admission, 1-year MACEs, and 1-year CV mortality rate.

## Introduction

Despite the advancement and innovation of pharmacological and interventional therapies, the socioeconomic burden (morbidity, mortality, financial cost) of myocardial infarction (MI) remains high worldwide (1, 2). The situation is of no exception in China, where the number of ischemic heart disease events substantially increased from 0.75 million in 1990 to 1.4 million in 2013 (3). In 2010, approximately one million deaths were caused by MI (4). In addition, the annual growth rate of the total hospitalization cost of MI has been 26% since 2004 and reached 177 billion CNY alone for the entire hospitalization cost of cardiovascular (CV) diseases (5, 6). A method that is preferably inexpensive, easily accessible, and does not require medical supervision may reduce the severity and improve the survival of MI patients and is thus clinically relevant.

The World Health Organization published an updated guideline on habitual physical activity (HPA), which recommends that all adults perform at least 75–150 min of vigorous-intensity aerobic physical activity per week (7). Evidence has shown that HPA can improve the prognosis of end-stage renal disease, asthma, and heart valve disease (8–10) as well as reduce dementia (11). Despite some studies that highlighted the benefits of HPA after MI (7), the effect of HPA before the onset of MI is unclear.

The purpose of our study was first to evaluate whether HPA was associated with the severity of MI on admission and further sought to determine whether HPA could reduce major adverse cardiovascular events (MACEs), CV mortality, and cardiac-related readmission following MI.

## Methods

### Enrolment and allocation of patients

This retrospective study was conducted in accordance with the principles of the Declaration of Helsinki. The study was approved by the ethics committee of the hospital of which all patients provided written informed consent [code number: Ethic(2022)246]. The inclusion criteria were patients aged > 18 years admitted to the department of cardiology at the hospital between January 2018 and December 2020 with a diagnosis of ST-segment elevation myocardial infarction (STEMI) or non–ST-segment elevation myocardial infarction (NSTEMI) defined according to the latest guidelines (12). The exclusion criteria included those with any comorbid condition that limited HPA (e.g., leg amputation, advanced cancer, disabling stroke, Parkinson's disease) or severe mental illness that would interfere with the ability to engage in HPA.

Patients were allocated to the HPA or non-HPA group based on whether they engaged in HPA before the index admission. HPA is defined as moderate to vigorous activity, including walking, swimming, using a treadmill, cycling, and using an ergometer, tennis, and other kinds of physical activity, with a duration of no less than 150 min/weak (13).

### Data collection, follow-up assessments, and outcomes

Participants were asked to complete a questionnaire at baseline during their index admission. Clinical characteristics were extracted from medical charts, including demographic characteristics [age, sex, body mass index (BMI)], cardiovascular risk factors (smoking, alcohol consumption, hypertension, diabetes, family history of MI and interventional therapy), and blood biochemical index [cardiac troponin T (cTnT), low-density lipoprotein (LDL) and high-density lipoprotein (HDL)]. The Killip class during the index admission was ascertained by the cardiologist in charge according to the guideline as follows (14):

• Class I: No signs of heart failure, but PCWP (pulmonary capillary wedge pressure) may be elevated, with a fatality rate of 0%–5%

• Class II: mild to moderate heart failure, lung rates are less than 50% of the two lung fields, third heart sound, persistent sinus tachycardia or other arrhythmias, elevated venous pressure, x-ray manifestations of pulmonary congestion, with a mortality rate of 10%–20%

• Class III: severe heart failure with acute pulmonary edema, rates in more than 50% of both lungs, and a fatality rate of 35%–40%

• Class IV: cardiogenic shock, systolic blood pressure less than 90 mmHg, urine output less than 20 ml/h, cold and clammy skin, cyanosis, accelerated breathing, pulse rate more than 100 beats/min, and a case fatality rate of 85%–95%

All patients were followed up by phone and/or clinical visits to collect clinical outcome information, including 1-year MACEs (defined as death from any CV diseases, cardiac readmission for nonfatal MI, or nonfatal heart failure or arrhythmia within 1 year following the index date of admission), 1-year CV mortality (defined as death from any CV diseases within 1 year following the index date of admission), and 1-year cardiac-related readmission rate (defined as hospital admissions for nonfatal cardiac causes within 1 year following the index date of admission, which included reinfarction, heart failure, and arrhythmia).

### Statistical analysis

All analytical tasks were completed by using SPSS v24.0 (IBM Corp.). For follow-up assessments, the baseline was defined as the date the patient was first admitted to the hospital. To compare the differences in baseline demographics between those with and without HPA, continuous variables and categorical variables of the two groups were analyzed by Student's *t*-test and the chi-square (*χ*^2^) test, respectively. The age, BMI, LDL, and HDL results are described as the means and standard deviation (means ± SD), and the cTnT results are presented as the medians and interquartile ranges [medians (IQRs)], whereas the cardiovascular risk factors like smoking, alcohol consumption, hypertension, diabetes, family history of MI, and interventional therapy are presented as number and percentage. *p* ≤ 0.05 denoted significant differences between the two groups.

For analysis of the influence of HPA on the Killip class, we performed univariate and multivariate binary logistic regression analyses. The quantitative variables (age, BMI, and cTnT) and dichotomous variables (HPA, sex, smoking, alcohol consumption, hypertension, diabetes, family history of MI, and interventional therapy) identified in the univariate analysis as being significantly associated with a lower Killip class (*p* ≤ 0.05) were further analyzed with a multivariate binary logistic regression model. For analysis of the influence of HPA on 1-year MACEs, 1-year CV mortality, and 1-year cardiac readmission rate, we performed univariate and multivariate binary logistic regression analyses. The quantitative variables (age and BMI) and dichotomous variables (HPA, sex, smoking, alcohol consumption, hypertension, diabetes, family history of MI, interventional therapy, and higher Killip class) identified in the univariate analysis as being significantly associated with 1-year MACEs, 1-year CV mortality, and 1-year cardiac readmission rate (*p* ≤ 0.05) were further analyzed with a multivariate binary logistic regression model. Effect sizes are given as odds ratio (OR) and 95% confidence interval (95% CI). Interaction analysis was used to investigate the comprehensive effects of HPA and subgroup factors on the probability of adverse outcomes. Effect sizes are demonstrated as OR and 95% CI. Student's *t*-test was used for the comparison between continuous variables, and the *χ*^2^ test was used for the comparison between categorical variables. *p* ≤ 0.05 denoted significant differences between the two groups.

## Results

### Participants

**Figure 1:**
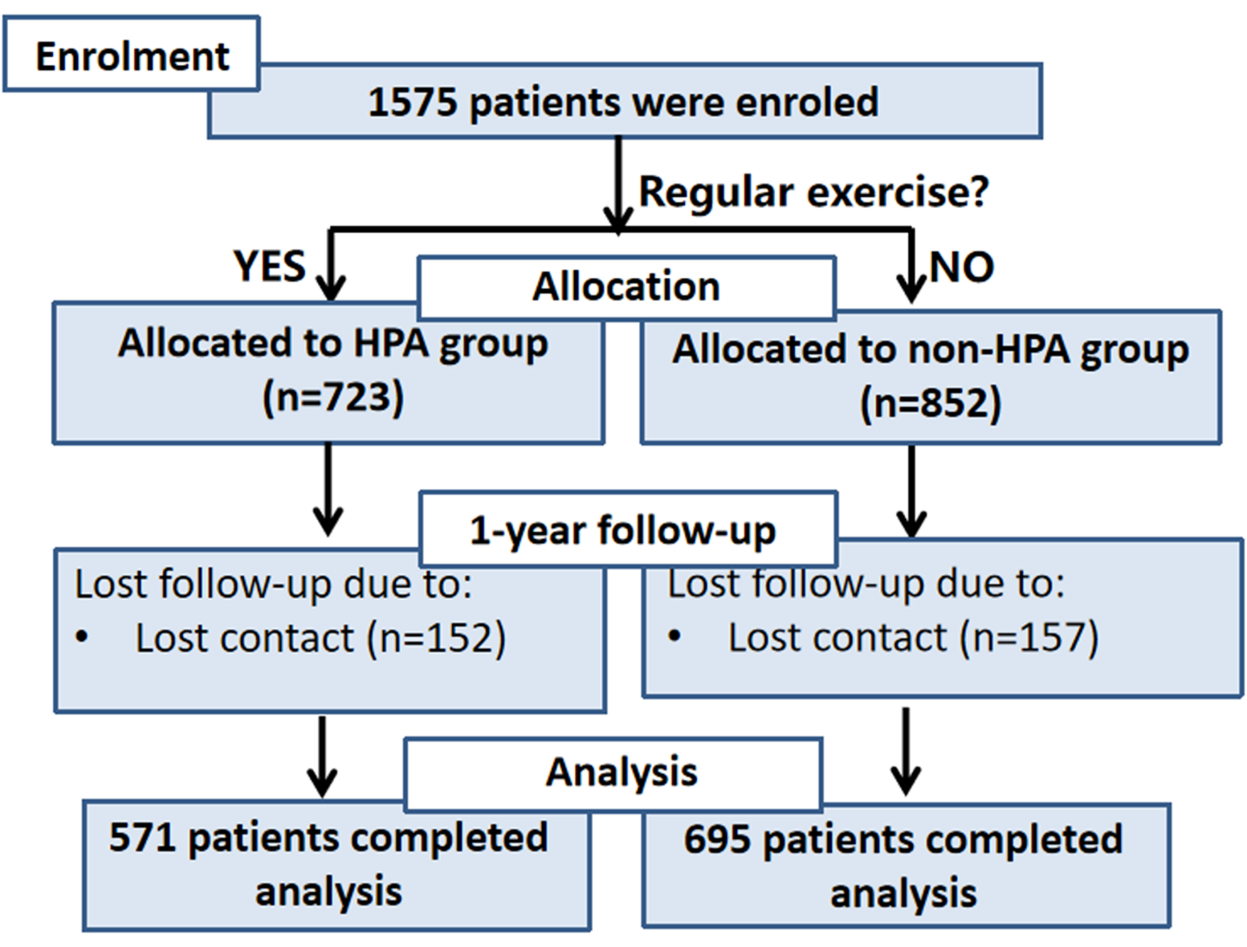
Flowchart of participants.

The study flow diagram is shown in Figure 1. Between January 2018 and December 2020, 1,575 patients were admitted for MI, and all gave written consent. Based on the information in the questionnaire and the definition of HPA mentioned above, 723 participants were considered to engage in HPA, while the rest of the 852 participants were allocated to the non-HPA group. Information about key outcomes, including 1-year MACEs, 1-year CV mortality, and 1-year cardiac-related readmission rate, was collected for all except 309 patients (152 patients with HPA and 157 patients without HPA) who were lost to follow-up during the 1-year follow-up period. Finally, 571 patients with HPA and 695 patients without HPA completed this 1-year follow-up study.

### Baseline demographic in the HPA and non-HPA groups

Baseline characteristics are shown in Table 1. We recruited an MI population of elderly, predominantly male patients (age, 63.4 ± 12.6 years; sex, 72% are male). Among the population, participants who engaged in HPA were younger (61.4 ± 11.5 years vs. 64.9 ± 13.3 years, *p* = 0.001), more likely to be male (77% vs. 68%, *p* = 0.001), consume alcohol (30% vs. 20%, *p* < 0.001), have a family history of MI (43% vs. 32%, *p* < 0.001), and receive interventional therapy (77% vs. 51%, *p* < 0.001) than those who did not engage in HPA. For the prevalence of smoking, hypertension, and diabetes, as well as BMI, cTnT, LDL, and HDL values, there were no differences between the HPA and non-HPA groups at baseline.

**Table 1:** Demographic and clinical characteristics at baseline.

^a^Characteristics	Overall (*n* = 1,266)	HPA group (*n* = 571)	Non-HPA group (*n* = 695)	*p* Value
**Demographic characteristics**				
Age (year)	63.4 ± 12.6	61.4 ± 11.5	64.9 ± 13.3	0.001
Male, *n* (%)	908 (72%)	435 (77%)	473 (68%)	0.001
BMI (kg/m^2^)	24.3 ± 3.6	24.5 ± 3.2	24.1 ± 3.8	0.380
**Cardiovascular risk factors**				
Smoking, *n* (%)	481 (38%)	217 (38%)	264 (38%)	0.995
Alcohol consumption, *n* (%)	308 (24%)	171 (30%)	137 (20%)	<0.001
Hypertension, *n* (%)	691 (55%)	309 (54%)	382 (55%)	0.763
Diabetes, *n* (%)	388 (31%)	160 (28%)	228 (33%)	0.066
^b^Family history of MI, *n* (%)	470 (32%)	246 (43%)	224 (32%)	<0.001
Interventional therapy, *n* (%)	890 (70%)	438 (77%)	452 (51%)	<0.001
**Blood biochemical index**				
cTnT (ug/L)	1.9 (0.3, 5.9)	1.8 (0.2, 5.7)	2.1 (0.4, 6.0)	0.355
LDL (mmol/L)	2.7 ± 1.2	2.7 ± 1.1	2.7 ± 1.2	0.816
HDL (mmol/L)	1.1 ± 0.3	1.1 ± 0.3	1.1 ± 0.3	0.091

BMI, body mass index; cTnT, cardiac troponin T; LDL, low-density lipoprotein; HDL, high-density lipoprotein.

^a^ Age, BMI, LDL, and HDL are presented as mean and standard deviation (mean ± SD); cTnT is presented as median and interquartile range, and the rest of the data are presented as number and percentage (*n*, %).

^b^ Family history of MI is defined as hospitalization due to myocardial infarction, angina with coronary revascularization, or cardiovascular death in ≥1 parent or full sibling, with early onset defined as disease onset before 55 years for men and 65 years for women.

### HPA was associated with a lower Killip class

**Figure 2:**
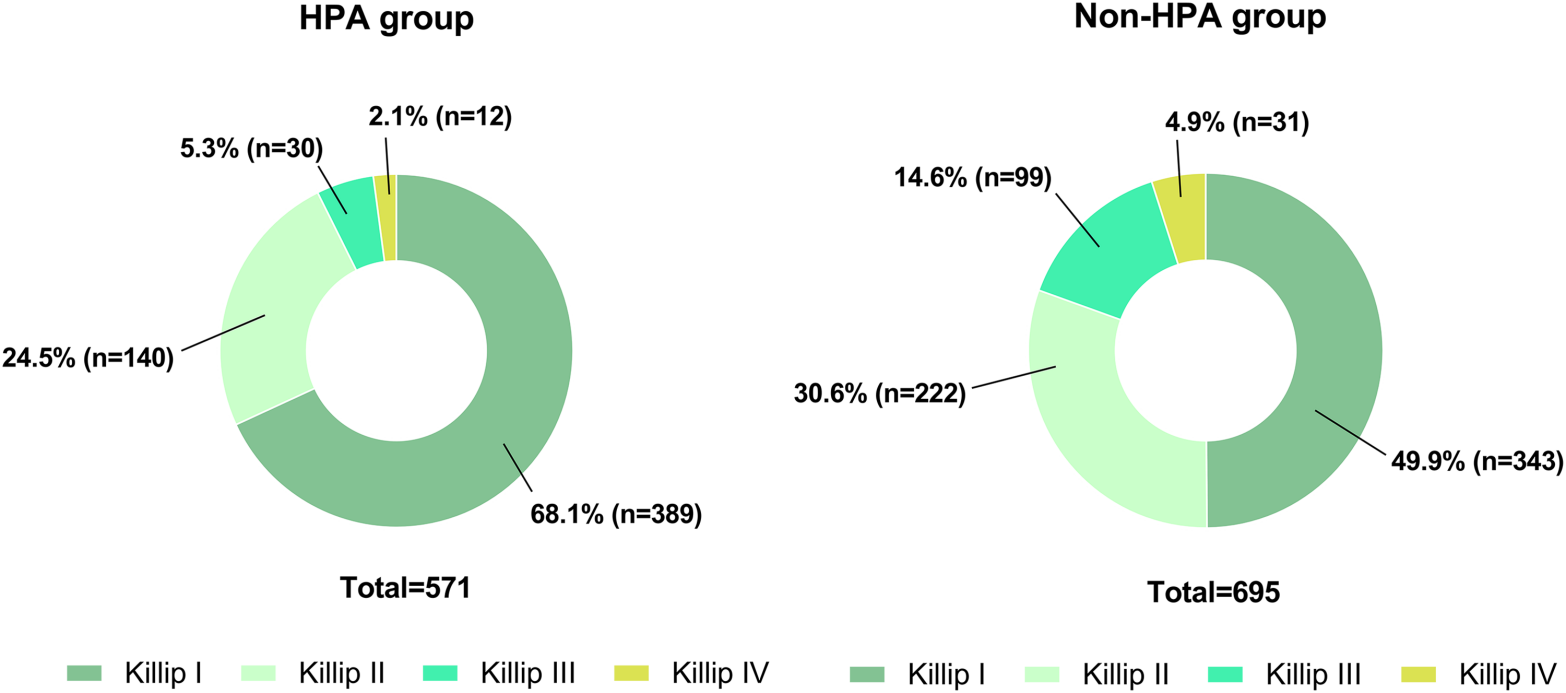
The pie chart demonstrates the distribution of patients with different Killip classes in the two groups on admission. (**A**) The number and the ratio of patients with different Killip classes in the HPA group (*n* = 571). (**B**) The number and the ratio of patients with different Killip classes in the non-HPA group (*n* = 695).

According to the Killip classification, 732 (58%) patients were in class I, 362 (29%) were in class II, 129 (10%) were in class III, and 43 (3%) were in class IV upon admission. The distribution of participants in different Killip classes among patients who engaged in HPA and those who did not are depicted in Figure 2. Patients who engaged in HPA were less likely to be in Killip class III (5% vs. 15%) and Killip class IV (2% vs. 5%) upon admission to MI than those who did not engage in HPA.

The relationship between a high Killip class (Killip classes III and IV) and HPA is shown in Table 2. Univariate analysis demonstrated that a higher Killip class was negatively associated with HPA (OR = 0.35: 95% CI, 0.24–0.50; *p* < 0.001), which remained significant following multivariate adjustment for sociodemographic factors (age, sex, smoking, and alcohol consumption) and clinical demographic factors (hypertension, diabetes, family history of MI, interventional therapy, BMI, and cTnT) (adjusted OR = 0.48: 95% CI, 0.32–0.71; *p* < 0.001).

**Table 2:** Effect of HPA on higher Killip class.

	Higher Killip class (Killip classes III and IV)					
	Univariate			Multivariate^a^		
	OR	95% CI	*p* Value	OR	95% CI	*p* Value
HPA	0.35	0.24–0.50	<0.001	0.48	0.32–0.71	<0.001
Age	1.06	1.05–1.08	<0.001	1.04	1.02–1.06	<0.001
Male	0.52	0.37–0.73	<0.001	0.91	0.61–1.37	0.642
Smoking	0.47	0.32–0.67	<0.001	0.77	0.49–1.21	0.293
Alcohol consumption	0.32	0.20–0.54	<0.001	0.63	0.35–1.11	0.101
Hypertension	1.36	0.98–1.89	0.068	–^b^	–	–
Diabetes	2.22	1.60–3.08	<0.001	2.16	1.52–3.07	<0.001
Family history of MI	0.99	0.77–1.28	0.947	–	–	–
Interventional therapy	0.45	0.32–0.61	<0.001	0.59	0.41–0.84	0.003
BMI	0.88	0.84–0.93	<0.001	0.93	0.88–0.98	0.005
cTnT	1.09	0.77–1.55	0.616	–	–	–

OR, odds ratio; 95% CI, 95% confidence interval; HPA, habitual physical activity; MI, myocardial infarction; BMI, body mass index; cTnT, cardiac troponin T.

^a^ Adjusted for the factors whose *p* value is less than 0.05 in univariate regression analysis.

^b^ The dash “–” indicates the factors did not involve in the multivariate regression analysis.

### HPA independently associated with 1-year MACEs

Variables related to 1-year MACEs, including HPA, age, sex, smoking, alcohol consumption, hypertension, diabetes, family history of MI, interventional therapy, BMI, and higher Killip class, are described in Table 3. Univariate analysis demonstrated that patients who had HPA had significantly fewer 1-year MACEs than those who were inactive (OR = 0.57: 95% CI, 0.44–0.74; *p* < 0.001), which remained significant following multivariable adjustment (adjusted OR = 0.74: 95% CI, 0.56–0.98; *p* = 0.038).

**Table 3:** Effect of HPA on 1-year MACEs.

	1-year MACEs					
	Univariate			Multivariate^a^		
	OR	95% CI	*p* Value	OR	95% CI	*p* Value
HPA	0.57	0.44–0.74	<0.001	0.74	0.56–0.98	0.038
Age	1.06	1.05–1.07	<0.001	1.04	1.03–1.05	<0.001
Male	0.51	0.39–0.67	<0.001	0.98	0.71–1.36	0.910
Smoking	0.47	0.36–0.63	<0.001	0.70	0.50–0.99	0.041
Alcohol consumption	0.50	0.36–0.69	<0.001	0.95	0.65–1.40	0.800
Hypertension	1.12	0.87–1.44	0.376	–^b^	–	–
Diabetes	1.40	1.07–1.82	0.013	1.28	0.96–1.71	0.087
Family history of MI	1.04	0.85–1.27	0.717	–	–	–
Interventional therapy	0.44	0.34–0.57	<0.001	0.56	0.42–0.74	<0.001
BMI	0.90	0.87–0.94	<0.001	0.95	0.91–0.99	0.010
^c^Higher Killip class	5.07	3.62–7.09	<0.001	3.34	2.33–4.80	<0.001

OR, odds ratio; 95% CI, 95% confidence interval; HPA, habitual physical activity; MI, myocardial infarction; BMI, body mass index.

^a^ Adjusted for the factors whose *p* value is less than 0.05 in univariate regression analysis.

^b^ The dash “–” indicates that the factors did not involve in the multivariate regression analysis.

^c^ Higher Killip class indicates the participants who are in Killip class III or IV.

**Figure 3:**
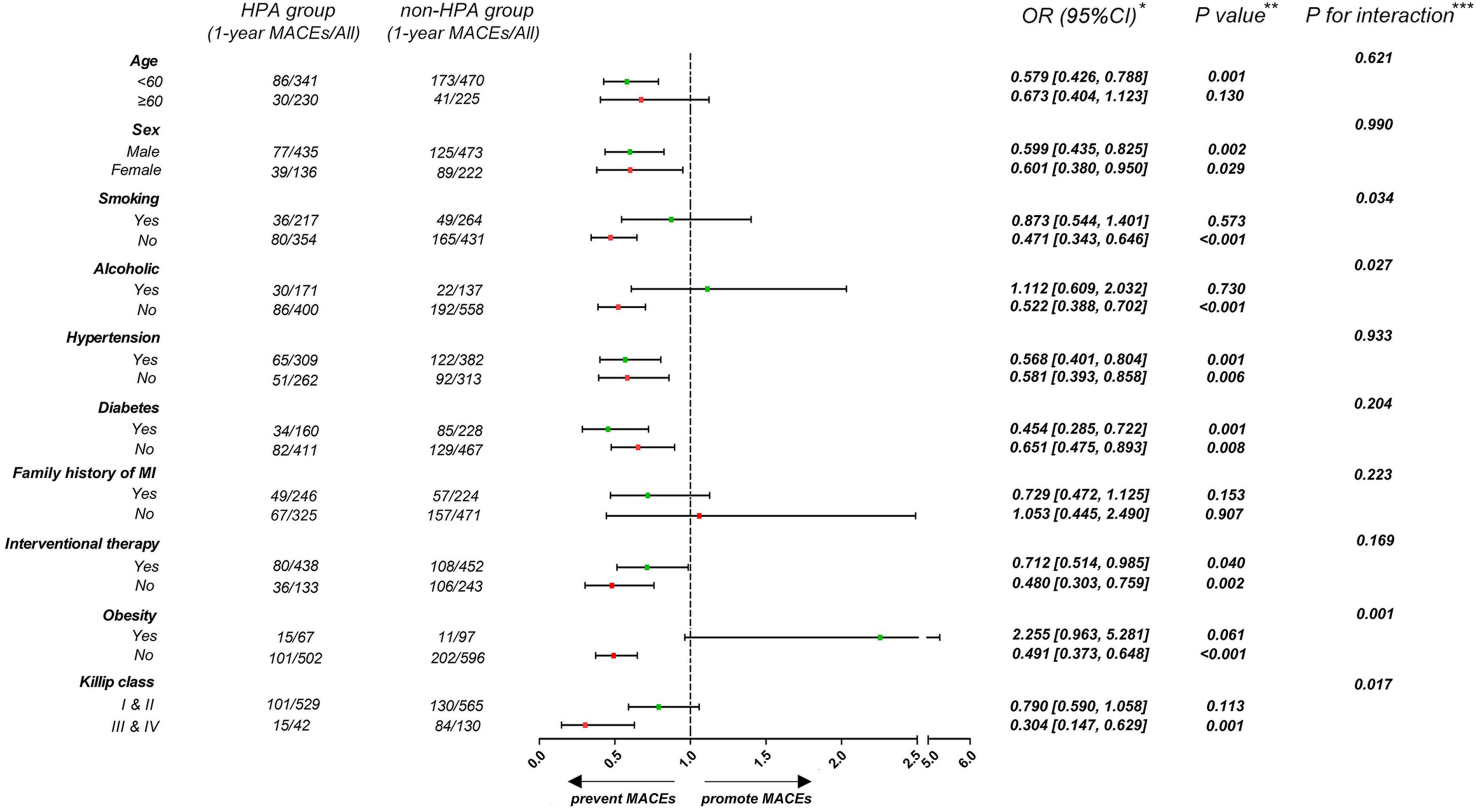
Forest plot demonstrates the effect of HPA on 1-year MACEs in different subgroups. *Data are presented as OR (95% CI) to interpret the effect sizes of HPA on 1-year MACEs in subgroups. **P values are reported for the difference of effect sizes on 1-year MACEs between HPA and non-HPA group. ***P for interaction are reported for interaction effect of HPA and subgroup factors.

To investigate the influence of HPA on 1-year MACEs in different subgroups, patients were stratified according to age (<60 or ≥60 years), sex (male or female), history of smoking, alcohol consumption, hypertension and diabetes (presence or absence), family history of MI (presence or absence), interventional therapy (presence or absence), obesity (BMI < 28 or ≥28 kg/m^2^), and Killip class (higher or lower) (Figure 3). The association of HPA and a lower risk of 1-year MACEs following MI was consistent for both sexes, irrespective of history of hypertension, diabetes, or interventional therapy (*p* for interaction >0.05). The effect of HPA was more pronounced among those who did not smoke or drink, were not obese, and had a higher Killip class (*p* for interaction <0.05).

### HPA independently associated with 1-year CV mortality

We further investigated the effect of HPA on 1-year CV mortality and 1-year cardiac readmission rate. As shown in Table 4, participants with HPA had a lower risk of 1-year CV mortality after multivariate adjustment (OR = 0.50: 95% CI, 0.28–0.88; *p* = 0.017). Nonetheless, there was no significant association between HPA and the 1-year cardiac readmission rate (OR = 0.87: 95% CI, 0.64–1.17; *p* = 0.346) (Supplementary Table S1).

**Table 4:** Effect of HPA on 1-year CV mortality.

	1-year CV mortality					
	Univariate			Multivariate^a^		
	OR	95% CI	*p* Value	OR	95% CI	*p* Value
HPA	0.23	0.14–0.37	<0.001	0.50	0.28–0.88	0.017
Age	1.12	1.10–1.15	<0.001	1.08	1.05–1.11	<.001
Male	0.58	0.39–0.86	0.007	0.54	0.31–0.95	0.033
Smoking	0.33	0.20–0.52	<0.001	0.57	0.29–1.10	0.091
Alcohol consumption	0.14	0.06–0.33	<0.001	0.35	0.13–0.93	0.036
Hypertension	1.07	0.73–1.07	0.739	–^b^	–	–
Diabetes	1.26	0.84–1.88	0.272	–	–	–
Family history of MI	1.25	0.91–1.72	0.167	–	–	–
Interventional therapy	0.14	0.09–0.21	<0.001	0.22	0.13–0.37	<0.001
BMI	0.82	0.77–0.87	<0.001	0.93	0.87–1.00	0.049
Higher Killip class	13.76	8.93–21.20	<0.001	8.48	5.03–14.30	<0.001

OR, odds ratio; 95% CI, 95% confidence interval; HPA, habitual physical activity; MI, myocardial infarction; BMI, body mass index.

^a^ Adjusted for the factors whose *p* value is less than 0.05 in univariate regression analysis.

^b^ The dash “–” indicates that the factors did not involve in the multivariate regression analysis.

^c^ Higher Killip class indicates the participants who are in Killip class III or IV.

**Figure 4:**
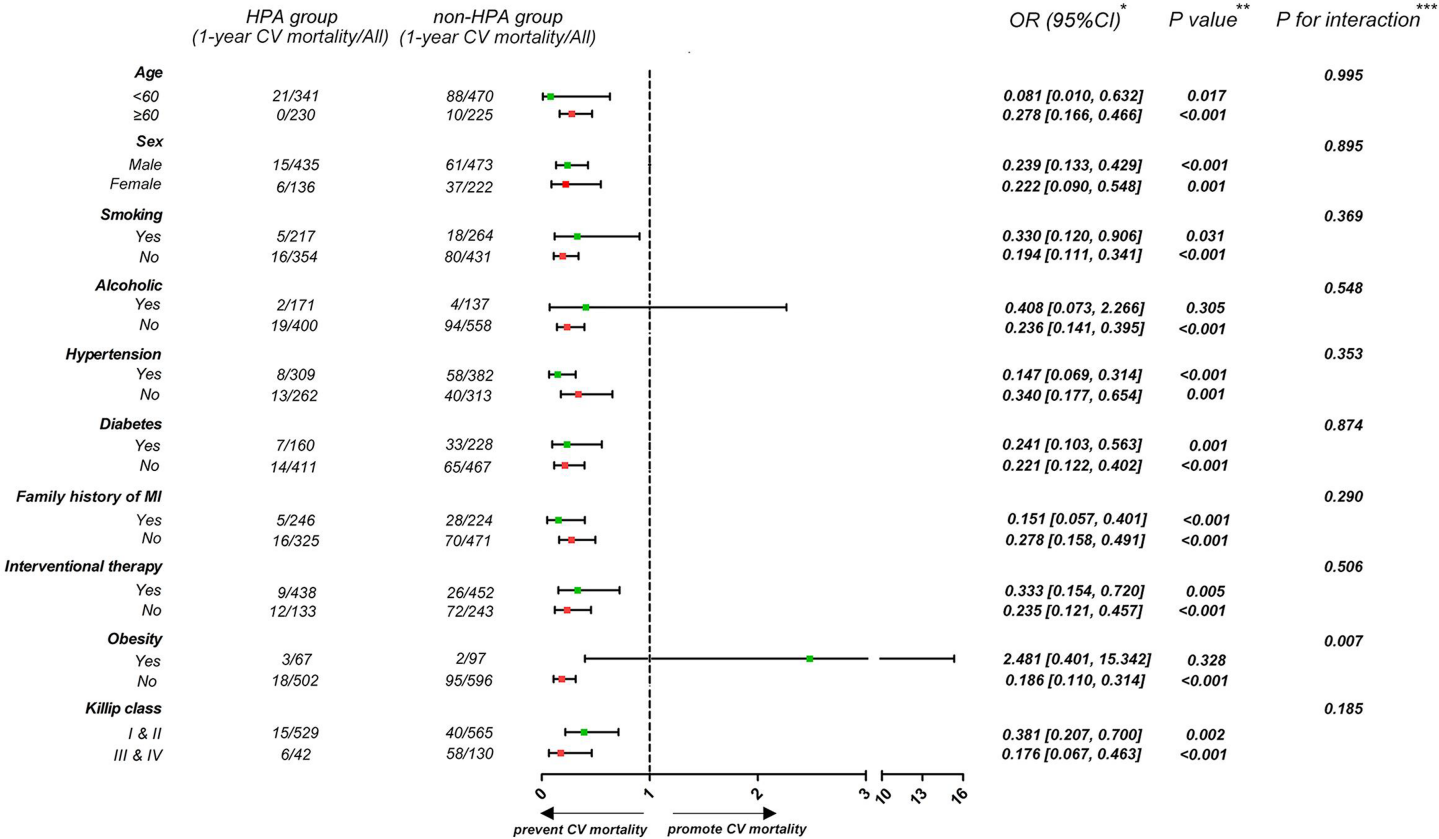
Forest plot demonstrates the effect of HPA on 1-year CV mortality in different subgroups. *Data are presented as OR (95% CI) to interpret the effect sizes of HPA on 1-year CV mortality in subgroups. **P values are reported for the difference of effect sizes on 1-year CV mortality between HPA and non-HPA group. ***P for interaction are reported for interaction effect of HPA and subgroup factors.

To investigate the influence of HPA on 1-year CV mortality among patients in different subgroups, participants were stratified according to age (<60 or ≥60 years), sex (male or female), history of smoking, alcohol consumption, hypertension and diabetes (presence or absence), family history of MI (presence or absence), interventional therapy (presence or absence), obesity (BMI < 28 or ≥28 kg/m^2^), and Killip class (higher or lower) (Figure 4). Patients who engaged in HPA had a lower rate of 1-year CV mortality irrespective of age, sex, history of smoking, hypertension and diabetes, family history of MI, interventional therapy, and Killip class (*p* for interaction >0.05). The effect of HPA on lower 1-year CV mortality was more apparent among non-obese patients than among those who were obese (*p* for interaction <0.01).

## Discussion

The present study demonstrated that patients with MI who engaged in HPA were more likely to have a lower Killip class upon admission, 1-year MACEs, and 1-year CV mortality than those who were physically inactive. The benefit of HPA for 1-year MACEs and 1-year CV mortality following MI was seen across various subgroups.

According to the 2008 Physical Activity Guidelines Advisory Committee (15) and the Centers for Disease Control and Prevention guideline (16), 150 min/week of moderate-intensity physical activity should be recommended to all because such physical activity provides substantial health improvements, including lower mortality and morbidity (17). In a systematic review and meta-analysis of 33 cohort studies that included 883 372 participants, HPA was associated with a 35% risk reduction for CV mortality and a 33% risk reduction for all-cause mortality (18). While studies have shown that exercise may reduce the incidence and mortality of CV diseases, no study has addressed the benefit of HPA (including CV disease severity upon admission, subsequent cardiac-related readmission, and mortality) for individuals who have MI. In this study, we reported that HPA evidently reduced the Killip class upon admission and the incidence of 1-year MACEs following MI. We further extended the benefit of HPA following MI by showing a reduction in 1-year CV mortality after multivariable adjustment.

Due to complex mechanisms and individual demographic differences, the clinical management of MI patients is clinically challenging. Of note, patient risk stratification is applied to establish therapeutic priorities for MI patients (19). Among the risk stratification models, the Thrombolysis in Myocardial Infarction (TIMI) risk model and the Global Registry of Acute Coronary Events (GRACE) risk score are the most commonly used models in clinical risk stratification and have excellent performance in diagnosis (20–22). However, current risk scores have not considered HPA as a potential risk factor (23, 24), despite it being a key prognosticator among MI patients. Our study not only verified that HPA is an independent factor associated with a less critical presentation (a lower Killip class) but also provided evidence that HPA predicts adverse clinical outcomes following MI. Whether the incorporation of HPA can improve risk stratification among patients with MI merits further evaluation.

There are multiple mechanisms by which HPA can improve outcomes in patients with MI. According to preliminary studies, MI patients benefited from HPA mainly by improving risk profiles, such as lowering triglyceride and increasing high-density lipoprotein cholesterol levels (25), lowering blood pressure (26), improving glucose metabolism and insulin sensitivity (27), and reducing BMI and inflammatory cytokines (28). The mechanisms mentioned above account for 59% of the reduction in the development of CV events (29). Patients can benefit further from HPA by other mechanisms, such as improved endothelial function (30), lowering sympathetic activity, and increased nitric oxide bioavailability (31), which contribute to the remaining 41% of the reduction in the development of CV events. Ischemic preconditioning *via* exercise may provide antioxidant fortification, prevent calcium dysregulation and improve bioenergetic reserve (32, 33). This evidence collectively supports that HPA provides CV protection in MI *via* multiple potential mechanisms.

In the current guidelines, HPA is recommended as the primary prevention measure against CV diseases (34, 35). In addition, HPA not only reduces overall mortality but also improves outcomes once MI/adverse events occur. It has been reported that long-term HPA can stop the progression and promote the regression of coronary atherosclerotic lesions (36). HPA may reduce the length of hospital stay for patients with ischemic heart diseases (37) and may lower the risk of vigorous activity-induced MI (38). Despite these advantages, only 50% of Americans follow these guidelines (13), and the average total HPA of Chinese adults showed a downward trend from 1991 to 2009. The average static behavior time of Chinese adults increased from 15.1 h/week in 1991 to 20.0 h/week in 2009 (39). Appropriate education for the general public heightens awareness of the potential benefit of HPA and encourages, in particular, those who are at risk of or with established ischemic heart diseases to implement habitual exercise, which is crucial to reduce the substantial health care burden due to MI.

The observational study was not possible to ascertain or adjust for all factors that may influence the effect of HPA on the outcomes of MI patients. We nonetheless accounted for a broad range of baseline comorbidities and clinical correlates with potential prognostic implications on MI in our analysis, which minimized the risk of residual confounding. To examine the benefit of HPA for MI patients, participants were asked to provide information about whether they engaged in HPA before they participated in this study. Due to substantial physical limitations following MI, we were not able to systematically collect information about HPA during follow-up for all patients.

## Conclusions

We demonstrated that patients with MI who engaged in HPA were associated with a lower Killip class upon admission, fewer 1-year MACEs, and a lower 1-year CV mortality rate than those who were physically inactive. Our findings emphasized that HPA may reduce adverse outcomes among patients who develop MI.

## Data Availability

The raw data supporting the conclusions of this article will be made available by the authors, without undue reservation.
