# Supplementary material for: Habitual physical activity improves outcomes among patients with myocardial infarction
Source: Front Cardiovasc Med. 2023 Jun 12;10:1174466. doi: 10.3389/fcvm.2023.1174466 (PMC10291190; doi:10.3389/fcvm.2023.1174466)
Supplement: Supplementary file 1 [file Table1.docx]

**Supplementary Table 1. Effect of HPA on 1-year Cardiac Readmission**

|  | 1-year cardiac readmission | | | | | |
| --- | --- | --- | --- | --- | --- | --- |
|  | Univariate | | | ^a^Multivariate | | |
|  | OR | 95% CI | *p* value | OR | 95% CI | *p* value |
| HPA | 0.87 | 0.64 - 1.17 | 0.346 | ^b^- | - | - |
| Age | 1.03 | 1.02 - 1.05 | < 0.001 | 1.02 | 1.01 - 1.04 | 0.006 |
| Male | 0.48 | 0.35 - 0.66 | < 0.001 | 0.71 | 0.49 - 1.03 | 0.074 |
| Smoking | 1.76 | 0.41 - 0.78 | 0.001 | 1.18 | 0.81 - 1.73 | 0.386 |
| Alcohol consumption | 0.74 | 0.52 - 1.06 | 0.099 | ^b^- | - | - |
| Hypertension | 1.15 | 0.85 - 1.56 | 0.357 | - | - | - |
| Diabetes | 1.48 | 1.09 - 2.03 | 0.013 | 1.25 | 0.91 - 1.73 | 0.175 |
| Family history of MI | 0.94 | 0.75 - 1.19 | 0.611 | - | - | - |
| Interventional therapy | 0.88 | 0.63 - 1.23 | 0.454 | - | - | - |
| BMI | 0.95 | 0.91 - 1.00 | 0.033 | 0.98 | 0.94 - 1.03 | 0.383 |
| Higher Killip class | 2.35 | 1.52 - 3.63 | < 0.001 | 1.80 | 1.14 - 2.83 | 0.011 |

Abbreviations: OR, odds ratio; 95% CI, 95% confidential interval; HPA, habitual physical activity; MI, myocardial infarction; BMI, body mass index.

^a^Adjusted for the factors whose *p* value less than 0.05 in univariate regression analysis.

^b^The dash “-” indicates that the factors did not involve in the multivariate regression analysis.

^c^ Higher Killip class indicates the participants who are in Killip class III or IV.
